# Can Strain Elastography Predict Malignancy of Soft Tissue Tumors in a Tertiary Sarcoma Center?

**DOI:** 10.3390/diagnostics10030148

**Published:** 2020-03-07

**Authors:** Jonathan Cohen, Iben Riishede, Jonathan Frederik Carlsen, Trine-Lise Lambine, Mikkel Seidelin Dam, Michael Mørk Petersen, Michael Bachmann Nielsen, Caroline Ewertsen

**Affiliations:** 1 Department of Diagnostic Radiology, Rigshospitalet, Blegdamsvej 9, 2100 Copenhagen OE, Denmark; jonathan.carlsen@gmail.com (J.F.C.); trine-lise.lambine@regionh.dk (T.-L.L.); mikkel.seidelin.dam@regionh.dk (M.S.D.); michael.bachmann.nielsen@regionh.dk (M.B.N.); caroline.ewertsen@regionh.dk (C.E.); 2 University of Copenhagen, Faculty of Health and Medical Sciences, Panum Institute, Blegdamsvej 3B, 2200 Copenhagen N, Denmark; 3 Department of Obstetrics and Gynaecology, Rigshospitalet, Blegdamsvej 9, 2100 Copenhagen OE, Denmark; ibenriishede@gmail.com; 4 Musculoskeletal Tumor Section, Department of Orthopedic Surgery, Rigshospitalet, Blegdamsvej 9, 2100 Copenhagen OE, Denmark; michael.moerk.petersen@regionh.dk

**Keywords:** elastography, sarcoma, soft tissue tumors, diagnostic ultrasound, strain ratio, Tsukuba elasticity score

## Abstract

This study aims to investigate the ability of ultrasound strain elastography as an adjunct to predict malignancy in soft tissue tumors suspect of sarcoma or metastasis in a tertiary reference center for sarcoma. A total of 137 patients were included prospectively. Patients were referred on the basis of clinical or radiological suspicion of malignant soft tissue tumor. All patients had previously undergone diagnostic imaging (MRI, CT or PET-CT). After recording strain elastography cine loops, ultrasound guided biopsy was performed. Three investigators, who were blinded to final diagnosis, reviewed all elastograms retrospectively. For each elastogram, a qualitative, visual 5-point score was decided in consensus and a strain ratio was calculated. Final pathology obtained from biopsy or tumor resection served as gold standard. Eighty-one tumors were benign, and 56 were malignant. t-tests showed a significant difference in mean visual score between benign and malignant tumors. There was no significant difference in mean strain ratio between the two groups. Strain elastography may be a valuable adjunct to conventional B-mode ultrasound, perhaps primarily in primary care, when considering whether to refer to a sarcoma center or to biopsy, although biopsies cannot reliably be ruled out based on the current data.

## 1. Introduction

Sarcomas are a heterogeneous group of malignant tumors, ranging over 80 different histological diagnoses [1]. The terminology is complex, but sarcomas are commonly grouped in two categories: bone sarcomas and soft tissue sarcomas (STS). Although there is some discrepancy in the literature as to which soft tissue tumors (STT) are categorized as STSs [2], STS remains a relatively rare diagnosis, with an estimated incidence averaging 4–5/100.000 per year in Europe (excluding gastro-intestinal stromal tumors). The relative 5-year survival rate is only around 60% [3]. When a musculoskeletal STT is suspected, ultrasound (US) is often the first examination performed. Conventional US as pre-biopsy imaging is, however, insufficient [1]. 

Akin to manual palpation, strain elastography (SE) examination is based on the notion that malignant tumors are stiffer than benign tumors [4]. Applying elastography in addition to conventional US examination to improve prediction of malignancy has been studied in many different organs, including breast [5,6,7], liver [8], and thyroid [9,10]. Conversely, few studies, with only relatively small sample sizes, have attempted to use SE to predict malignancy in musculoskeletal STTs [11,12,13,14]. 

SE visualizes relative differences in tissue strain, when mechanical pressure is applied. A strain ratio (SR) between one region of interest (ROI) in the mass and one ROI in adjacent reference tissue can be calculated to estimate the strain of the mass relative to the reference tissue, hereby semi-quantifying the stiffness of the mass [15]. In addition, results can be displayed as a color map overlaying the conventional B-mode image, with different colors corresponding to different strain levels relative to other tissues within the color overlay boundaries. This provides the clinician with a visual representation of relative strain inside the mass, which can be used to produce a visual scoring of the mass, potentially aiding differentiation between benign and malignant lesions. A specific scoring system has yet to be proposed for musculoskeletal STTs, but the Tsukuba Elasticity Score (TES) proposed by Itoh et al. for breast tumors [16] has been applied in a modified version to these lesions in the literature as well [11,13]. A qualitative, visual score of 1 to 5 is assigned to each tumor according to elastographic pattern and stiffness. Higher scores have been shown to be associated with higher risk of malignancy in STTs [11], breast [17] and thyroid [9] masses, and using TES to modify tumor grading has been proposed to prevent unnecessary biopsies in breast tumors [17].

A pilot study of 61 STTs by Riishede et al. showed that differentiation by SE between benign and malignant STTs may be more accurate when fat-containing tumors are excluded [13]. As information regarding fat content of the tumor is usually available from pre-biopsy diagnostic imaging, further investigation into whether the accuracy of SE can be improved when used exclusively for tumors without fat may be of clinical value.

Expanding on the aforementioned pilot study, this study aims to evaluate the ability of US SE as an adjunct to predict malignancy in STTs suspected to be STSs or metastatic lesions in a tertiary reference center for sarcomas. We wish to compare the accuracy of predictions based on SRs and visual scoring respectively. Finally, we aim to investigate whether the predictive strength of SE improves when fat-containing tumors are excluded.

## 2. Materials and Methods

### 2.1. Patients

All patients gave informed consent before participation. Written consent was waived. The National Danish Committee on Biomedical Research Ethics (Journal: H-2-2014-FSP1) approved the study (approval date 8 January 2014).

**Figure 1:**
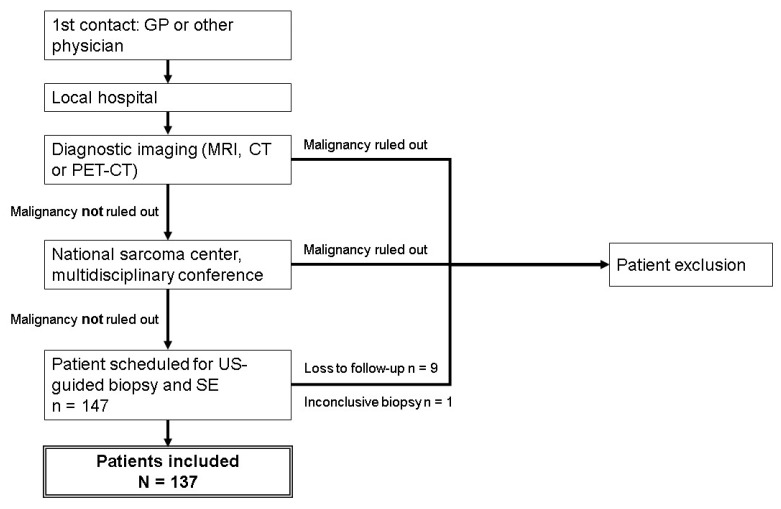
Patient inclusion. In total, 147 patients were screened for inclusion. Nine patients were lost to follow-up, and one patient had an inconclusive biopsy leading to exclusion. A total of 137 patients were included.

This study was a retrospective study of prospectively included patients. The study group consisted of patients referred to the sarcoma center at Rigshospitalet, Copenhagen University Hospital, Denmark. The center services 3.6 million people, and is the largest sarcoma center in Denmark. Patients were admitted between the 5th of May 2014 and the 9th of May 2016, and were referred on the basis of clinical or radiological suspicion of malignant STT. All referred patients had undergone diagnostic imaging (MRI, CT or PET-CT) at their local hospital before being evaluated at the multi-disciplinary tumor conference in our sarcoma center. Patients for whom malignancy could not be ruled out, and patients with inconclusive diagnostic imaging, were included and scheduled for US including elastography and subsequent US-guided biopsy. For patients with multiple tumors, only one tumor was included in the study, selected according to the best quality of US images and elastograms available. A total of 147 patients were included prospectively. Nine patients were subsequently excluded due to loss to follow-up, and one patient was excluded on the basis of an inconclusive histological diagnosis (Figure 1). The aforementioned pilot study by Riishede et al. was previously conducted in the same sarcoma center, but had no overlapping patients with the current study [13].

For patients who later underwent tumorectomy, final pathology served as the gold standard. For patients for whom tumorectomy was not indicated (patients with a benign biopsy), the histological biopsy was the gold standard.

### 2.2. Imaging and Biopsy

In all cases MRI, CT or PET-CT had been performed before referral to our center, and various imaging protocols had been used according to local set-ups. For most patients, at least 3 MRI sequences were available, including sequences after injection of an intravenous contrast agent. For patients with no available MRI, contrast-enhanced CT (2 patients) or PET-CT (8 patients) was used instead. Assessment hereof included tumor size, fat content, contrast enhancement and initial biopsy planning. Unavailability of MRI was due to either claustrophobia or contraindications to MRI. Some tumor borders were difficult to discern, and for these tumors, US was used to measure tumor size instead.

US was performed using a GE Logiq E9 system (GE Healthcare) with one of three probes (9L, ML6-15, C1-5) and preset according to anatomical location. B-mode US was performed to locate the tumor, and plan the biopsy entry point and track. Images showing the planned biopsy track and tumor size were stored for all patients.

**Figure 2:**
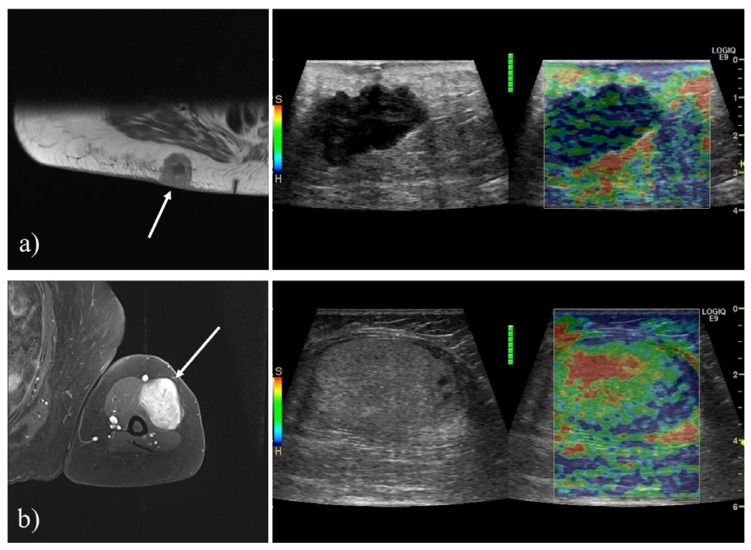
Examples of tumors included. (**a**) A 71-year-old female with metastasis of malignant melanoma. T1-weighted transverse contrast-enhanced MRI showing a lesion superficial to the right gluteal musculature. The tumor appeared hypoechoic and irregular on B-mode US, and SE revealed the tumor to be hard (blue). (**b**) A 75- year-old female with a chondroid lipoma. T1-weighted fat suppressed contrast enhanced MRI showing a tumor in the anterolateral upper arm. B-mode US and strain elastography (SE) revealed the tumor to be isoechoic, homogenous and soft (red).

All elastography examinations were performed by one of five different physicians, who all had extensive experience with the technique. Gentle compression was applied using the US transducer, at a frequency of 1.3–2 Hz, with continuous monitoring of elastogram quality using the quality indicator provided by the US system. Multiple 10-s elastography cine loops were stored. The color scale was adjusted to show stiff tissue as blue and soft tissue as red (Figure 2).

To ensure consistency, biopsies were then obtained from the exact location in which the elastography was performed. Biopsies preferentially targeted solid tumor areas with previously confirmed contrast enhancement. Biopsies were performed in local anesthesia (Lidocaine 10mg/mL) using an 18-gauge automatic biopsy needle (BARD). For most patients, at least two biopsies were taken to ensure a reliable diagnosis.

### 2.3. Ultrasound Evaluation

Stored US images and elastography cine loops were reviewed by three observers—two physicians with more than five years of experience in US, and a research fellow—who were all blinded to the patient medical history and final diagnosis.

**Figure 3:**
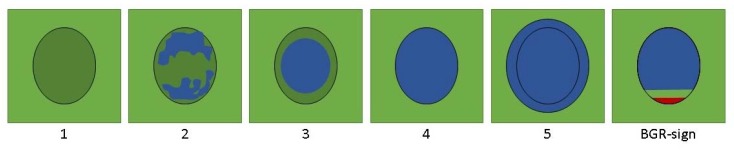
Tsukuba Elasticity Score (TES) scoring system after Itoh et al. and WFUMB guidelines by Barr et al. [16,17]. Elastographic patterns corresponding to a higher score are thought to be of higher risk of malignancy.

One elastography cine loop was chosen based on the quality and whether or not adequate adjacent reference tissue was visible. Using a modification of the Tsukuba Elasticity Score (TES) (Figure 3), a score was determined in consensus. Tumors displaying an elastographic pattern characteristic of a cystic lesion (BGR-sign) could not be given a numerical score [17].

**Figure 4:**
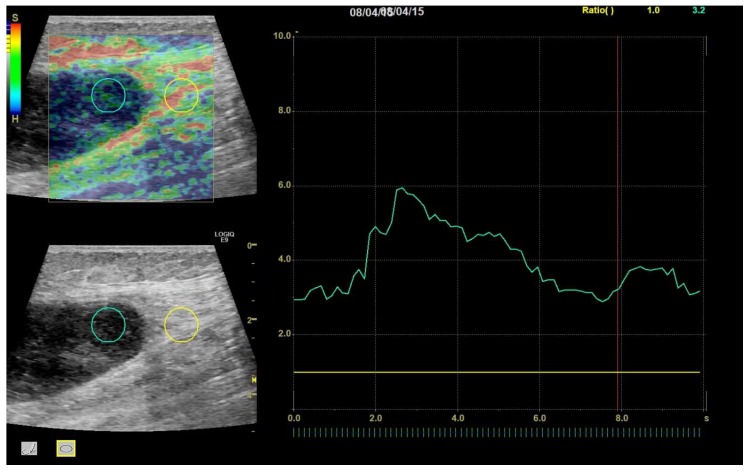
A 96-year-old female with myxoma. Region of interest (ROI) placement in tumor (left circle) and reference tissue (right circle). Tumor scored a TES of 4 and an average SR over the 10 s cine clip of 3.97.

Then, an SR was calculated for each frame by the ultrasound system from two circular or ovoid ROIs placed by the investigators—one tumor ROI inside the tumor and one reference ROI in nearby reference tissue. The tumor ROI was made as large as possible to ensure representability of the tumor, while avoiding obvious cystic areas, and while keeping a small distance to the tumor border to ensure that no reference tissue would be included. The reference ROI was placed outside the tumor boundary in nearby reference tissue at the same depth as the tumor, but tumor ROI and reference ROI were not necessarily of the same size, as ROI size has been shown to not affect SR [18]. Reference soft tissues were predominantly muscular, fatty, or connective tissues, and were selected to be of identical composition with the tumor where possible. For each clip, a mean SR for the entire 10 s clip was calculated (Figure 4).

Finally, the quality of the elastogram itself was scored according to how many frames for which the US system was able to produce an elastogram. For clips containing an elastogram in 0–25% of frames a score of 1 was given, 25–50% was given a score of 2, 50–75% was given a score of 3 and 75–100% was given a score of 4.

### 2.4. Statistical Analysis

Calculation and visualization of descriptive statistics and t-tests were done using Microsoft Excel 2013 (Microsoft, Redmond, WA, USA) and SPSS 25 (IBM, New York, USA). All t-tests were performed as two-tailed two-sample tests assuming unequal variances. The level of significance was set to *p* < 0.05.

For calculations using TES, tumors displaying BGR-sign were omitted, as they could not be given a meaningful numeric value for statistical analysis.

## 3. Results

**Figure 5:**
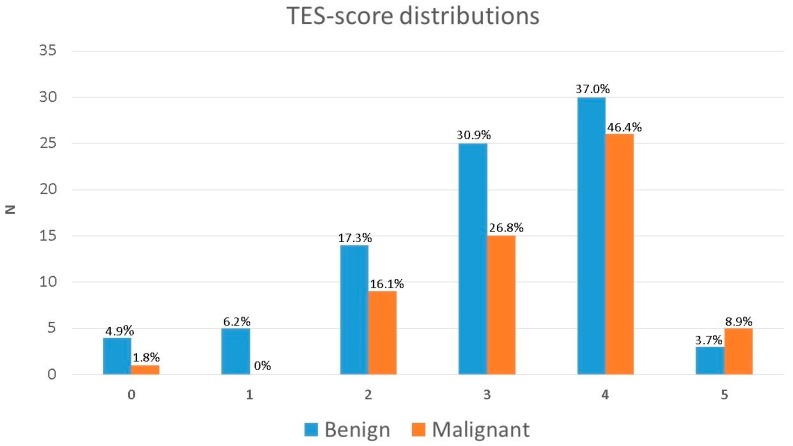
Distribution of TES-scores for benign and malignant tumors, respectively.

Of the 137 tumors examined in the study, 81 were benign, and 56 were malignant (Table 1). t-tests showed a significant difference between mean TES for malignant and benign tumors (3.16 and 3.49, respectively; *p* = 0.043), although it should be noted, that the CIs at the 95% level overlapped slightly (see Table 2; Table 3). The distribution of TESs is shown in Figure 5.

Predicting all tumors scoring TES 4 and above to be malignant would yield a sensitivity and specificity of 56% and 57%, respectively. Using TES 3 and above as the cut-off would raise sensitivity to 84%, but lower specificity to 24%.

The mean SR for all benign tumors was 2.30 (95% CI (1.86; 2.74)) and for all malignant tumors the mean SR was 2.66 (95% confidence interval (CI) (2.15; 3.16)). There was no statistically significant difference between the two means (*p* = 0.30).

Table 3 shows the mean TES with 95% CI for each pathological diagnosis. Of the 81 benign tumors, 32 (40%) contained fat, and of the 56 malignant tumors, 5 (9%) contained fat.

When fat-containing tumors were excluded, the mean SR for benign tumors was 2.68 (95% CI (2.03; 3.34)) and 2.75 (95% CI (2.2; 3.29)) for malignant tumors. There was no statistically significant difference between the two means (*p* = 0.88). When excluding fat-containing tumors, no significant difference in mean TESs could be found either (3.55 and 3.54; *p* = 0.92).

There were 18 (21%) benign tumors that had an SR above the upper limit of the CI of benign tumors. These were 4 instances of fibroma/fibromatosis, 4 schwannomas, 3 lipomas, 2 leiomyomas, 2 myxomas, 1 benign tumor of bone and cartilage (synovial chondromatosis), 1 benign vascular tumor (hemangioma), and 1 instance of non-specified necrotic debris.

Conversely, 32 (57%) of malignant tumors had an SR lower than the lower limit of the malignant tumor CI. These were 9 lymphomas, 4 myxofibrosarcomas, 3 pleomorphic sarcomas, 3 dedifferentiated liposarcomas, 2 myxoid liposarcomas, 2 osteosarcomas, 1 gastro-intestinal stromal tumor, 1 metastasis of hepatocellular carcinoma, 1 chondrosarcoma, 1 leiomyosarcoma, 1 highly differentiated liposarcoma, 1 metastasis of malignant melanoma, 1 metastasis of squamous cell carcinoma, 1 metastasis of unknown carcinoma and 1 unclassified sarcoma.

The only CI for TES that had no overlap with another CI, was the fibroma/fibromatosis category, which did not overlap with lipomas or with inflammation/non-specific reactive change. The fibroma/fibromatosis category did, however, overlap with all other categories, including the malignant tumors.

There was a significant difference in mean size (*p* = 0.007) between benign and malignant tumors.

Most elastograms were rated of the highest quality, with 90% of all elastograms rated quality 3 or 4. The mean quality score of all elastograms was 3.54.

## 4. Discussion

This study is, to our knowledge, the largest of its kind to evaluate the ability of SE to predict malignancy in soft tissue masses in a tertiary sarcoma center. We examined 137 tumors, and found a significant difference in mean TES between benign and malignant STTs.

The application of elastography has been studied in various musculoskeletal applications, and the 2018 EFSUMB guidelines for the clinical practice of elastography in non-hepatic applications has several recommendations for the use of musculoskeletal elastography in tendino- and neuropathic- conditions [19]. Still, no guidelines are currently available for the use of elastography in musculoskeletal cancer diagnostics. Furthermore, of the currently published studies, many use quantitative shear wave elastography instead of strain elastography, with methods not directly comparable to those of the current study [20].

Strain elastography, including TES is, however, well-validated in breast and thyroid tumors [9,17,21], which are generally more homogenous than the tumors included in this study. Applying TES to musculoskeletal lesions therefore proved non-trivial, underlining the importance of having all elastographies and the interpretations hereof in our study performed by radiologists with extensive experience with the technique.

In breast cancer, TES has been shown to have a sensitivity of 87–93% and a specificity of 86–90%, when using 4 and above as the cut-off for suspected malignancy [5]. We found considerably lower sensitivity and specificity when applying the same hypothetical cut off values to the tumors in this study, and so, defining an acceptable cut off value for which TES should elicit or exclude a biopsy seems improbable from the current data. Although there was a significant difference in mean TES between benign and malignant tumors, TES confidence intervals for benign and malignant tumors overlapped, suggesting that TES-distributions were too similar between the two groups for them to be reliably distinguished by TES alone.

We found no significant difference in SRs between benign and malignant tumors. This may partly be explained due to the within-group variability of SRs in both malignant and benign tumors. Over half of malignant tumors had an SR lower than the lower limit of the 95% CI for malignant tumors. We anticipated these soft but malignant tumors to be predominantly fat-containing tumors, but this was not clear-cut, as there was still no significant difference in mean SR when excluding fat-containing tumors, as suggested by a previous study [13]. Furthermore, a substantial amount of the benign tumors had an SR over the upper limit of the 95% CI, meaning they appeared harder than expected, and 3 of these were even benign lipomas, which are expected to be soft. Part of this may be explained by the elastography system assuming linear elastic behavior when calculating the tissue stiffness, when some tissues in reality may exhibit non-linear properties, especially at higher strain levels [4,22]. We attempted to mitigate this by ensuring elastograms were of proper quality, including rate of compression, as indicated by quality indicator of the US system.

**Figure 6:**
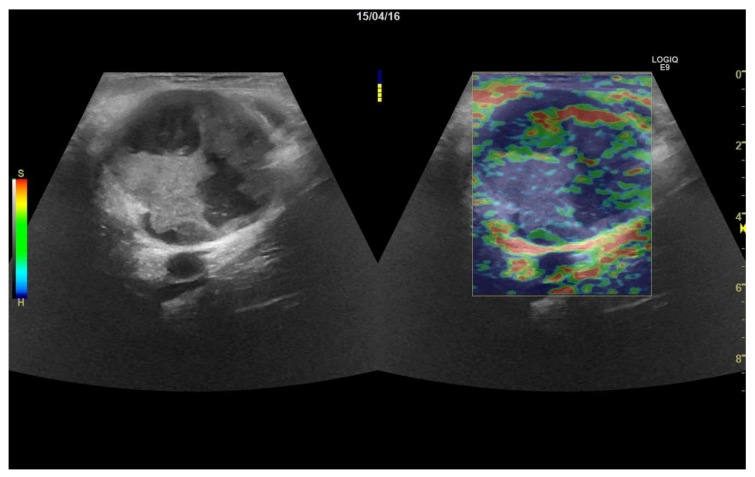
A 74-year-old female with metastasis of malignant melanoma. Ultrasound shows a highly heterogenous tumor, with both soft (red) and hard (blue) areas. The tumor was given a TES of 4 and a SR of 1.44.

The aforementioned heterogeneity of STTs and of musculoskeletal reference tissue complicates the use of SRs, as the selection of tumor- and reference ROI highly affects the calculated SR [23] (Figure 6). Furthermore, for 15 tumors, there was no useful reference tissue available at the exact depth of the tumor, which has been shown to affect the calculated SR [18]. Additionally, the histological composition of reference tissues varied, with some tumors being compared to fatty tissue, some to muscle and some to connective tissues. This was not possible to control for, as not all tumors were surrounded by the same types of tissues. Splitting the study group according to reference tissues would introduce bias, as tumors compared to fatty tissues would all appear hard, and tissues compared to muscles and tendons would vary uncontrollably according to the tension and stiffness of the muscle [24]. As there are still no guidelines available, we chose instead to adhere to the same reference tissue selection criteria as the all the previous comparable studies [11,13].

These challenges may explain why TES outperformed SR, as TES considers the entirety of the tumor elastogram rather than just a selected ROI. A visual scoring system specifically designed for STTs and proper standardization in selection of reference tissue, probe and US settings may prove useful in order to take into account the heterogeneity of both tumor and reference tissue.

Other studies have, however, managed to find significant differences between benign and malignant mean SRs, but to our knowledge, only two comparable previous studies exist. The previously mentioned pilot study by Riishede et.al. found a significant difference in a sample of 61 patients [13] and a study by Hahn et al. found a significant difference in a sample of 73 patients [11]. Because of these promising results, the current study attempted to establish the findings in a much larger sample, which, for SR, we were not able to. Interestingly, the only major difference in the methodology of these studies, when compared to the current study, seems to be the sample size, which, in the current study, is larger than that of the aforementioned two studies combined.

There were some limitations to our study. Firstly, we solely included patients referred to our tertiary sarcoma center. Among these patients, malignant tumors are vastly overrepresented, and regular lipomas are underrepresented when comparing to tumors found in primary diagnostic clinics [25]. The estimation of benign tumor average TES and SR may therefore be higher in our study than in the general population, resulting in a smaller difference in measured tumor stiffness between benign and malignant lesions. This would, in turn, result in an underestimation of elastography prediction strength. Furthermore, the patients in this study were included only after diagnostic imaging and MDT-presentation yielded no reliable diagnosis, resulting in only the most ambiguous and diagnostically challenging tumors being included. This could mean that SE may be of greater value in primary care, where US is used for diagnostics, and is not limited to biopsy guidance, which was the case in this study. Including only patients referred to our sarcoma center did however provide an unprecedented number of malignant tumors to study, ensuring a wide variety and high number of rare, malignant diagnoses.

Secondly, all imaging findings were evaluated retrospectively with reviewers blinded to the patients’ medical history and previous radiological findings. This eliminates potential bias in the evaluation of the elastogram itself, but may also lead to underestimation of the real effect of using SE as an adjunct in a clinical situation where this information is available to the radiologist/sonographer.

Additionally, we did not estimate inter-intra-observer variation. Because strain elastography in musculoskeletal STT applications is still so scarcely researched, there is no consensus for how to visually score STTs, how to produce elastography cine loops or select ROIs. None of these steps are trivial, and require careful consideration and experience, traits the authors decided to pool between the researchers by evaluating all images in consensus.

Finally, as SE is displayed as a color map overlaying the traditional B-mode image, we were unable to blind reviewers to B-mode image findings before evaluation of elastograms. This potentially biased reviewers according to the B-mode characteristics of the tumor. On the other hand, as the B-mode image is always visible in SE, this information would be present in a real, clinical setting as well.

In conclusion, we found that the mean TES of malignant tumors was significantly higher than that of benign tumors. SE may be a valuable adjunct to traditional B-mode US, perhaps primarily in primary diagnostics, when considering whether to refer for biopsy, however, biopsies cannot reliably be ruled out based on the current data, and further research and standardization is needed. We found no significant differences in SR between benign and malignant tumors.

## Figures and Tables

**Table 1:** Patient and tumor characteristics.

Characteristics	Benign	Malignant	Total
*n*, % of total	81 (59%)	56 (41%)	137
Male, % of total	48 (60%)	32 (40%)	80 (58%)
Female, % of total	33 (58%)	24 (42%)	57 (42%)
Fat-containing, % of benign/malignant	32 (40%)	5 (8.9%)	37 (27%)
Depth, mean in cm (±SD)	1.95 (±1.24)	2.58 (±1.32)	2.21 (±1.3)
Size, median in cm (range)	5.5 (1–30)	6.55 (1–34)	5.80 (1–34)

**Table 2:** Mean strain ratio (SR) and TES.

Tumor Category	SR		TES	
	Mean	*p*-Value	Mean	*p*-Value
Benign	2.30	0.30	3.16	0.043 *
Malignant	2.66		3.49	
Benign without fat	2.68	0.88	3.56	0.92
Malignant without fat	2.75		3.54	

* Statistically significant at the *p* < 0.05 level.

**Table 3:** Summary of diagnoses, and their respective TES.

Benign Tumors	*n*	Mean TES (±SD)	TES 95% CI
Lipoma	30	2.57 (±0.97)	(2.22; 2.91)
Inflammation/nonspecific reactive changes	8	2.88 (±0.35)	(2.63; 3.12)
Myxoma	8	3.86 (±1.41)	(2.81; 4.9)
Schwannoma	8	4 (±1.51)	(2.88; 5.12)
Benign vascular tumor	7	3.40 (±1.81)	(1.81; 4.99)
Fibroma/fibromatosis	6	3.83 (±0.75)	[3.23; 4.44)
Cyst	3	3.67 (±1.15)	(2.36; 4.97)
Benign tumors of bone and cartilage	2	4.00 (±0)	-
Other	9	3.22 (±1.09)	(2.51; 3.94)
Total	81	3.16 (±0.99)	(2.94; 3.38)
Total fat-containing	32	2.59 (±1.01)	(2.24; 2.94)
Total non-fat-containing	49	3.49 (±0.76)	(3.27; 3.71)
Malignant tumors	***n***	**Mean TES (±SD)**	**TES 95% CI**
Sarcoma	30	3.33 (±0.92)	(3.00; 3.66)
Metastasis	12	3.42 (±1.24)	(2.68; 4.15)
Lymphoma	11	3.73 (±0.90)	(3.19; 4.26)
Gastro-intestinal stromal tumor	1	4.00	-
Multiple myelomatosis	1	4.00	-
Malignant solitary fibrous tumor	1	2.00	-
Total	56	3.49 (±0.88)	(3.26; 3.72)
Total fat-containing	5	3.00 (±1.00)	(2.12; 3.88)
Total non-fat-containing	51	3.54 (±0.86)	(3.30; 3.78)
